# Hemodialysis in MNGIE transiently reduces serum and urine levels of thymidine and deoxyuridine, but not CSF levels and neurological function

**DOI:** 10.1186/s13023-017-0687-0

**Published:** 2017-08-01

**Authors:** Benjamin Röeben, Justus Marquetand, Benjamin Bender, Heiko Billing, Tobias B. Haack, Iciar Sanchez-Albisua, Ludger Schöls, Henk J. Blom, Matthis Synofzik

**Affiliations:** 1 Department of Neurodegeneration, Hertie Institute for Clinical Brain Research (HIH), University of Tübingen, 72076 Tübingen, Germany; 2 German Center for Neurodegenerative Diseases (DZNE), 72076 Tübingen, Germany; 3 Department of Epileptology, Hertie Institute for Clinical Brain Research (HIH), University of Tübingen, 72076 Tübingen, Germany; 4 Department of Neuroradiology, University of Tübingen, 72076 Tübingen, Germany; 5 Department of Child Nephrology, University Children’s Hospital Tübingen, 72076 Tübingen, Germany; 6 Institute of Human Genetics, Technische Universität München, 81675 Munich, Germany; 7 Institute of Human Genetics, Helmholtz Zentrum München, 85764 Neuherberg, Germany; 8 Institute of Medical Genetics and Applied Genomics, University of Tübingen, 72076 Tübingen, Germany; 9 Department of Pediatric Neurology and Developmental Medicine, University Children’s Hospital Tübingen, 72076 Tübingen, Germany; 10 Laboratory of Clinical Biochemistry and Metabolism, Department of General Pediatrics, Adolescent Medicine and Neonatology, University Medical Centre Freiburg, Freiburg, Germany

**Keywords:** MNGIE, Haemodialysis, Mitochondriopathy, Thymidine phosphorylases

## Abstract

Mitochondrial neurogastrointestinal encephalomyopathy (MNGIE) is a rare, autosomal-recessive mitochondrial disorder caused by *TYMP* mutations presenting with a multisystemic, often lethal syndrome of progressive leukoencephalopathy, ophthalmoparesis, demyelinating neuropathy, cachexia and gastrointestinal dysmotility. Hemodialysis (HMD) has been suggested as a treatment to reduce accumulation of thymidine and deoxyuridine. However, all studies so far have failed to measure the toxic metabolites in cerebrospinal fluid (CSF), which is the crucial compartment for CNS damage.

Our study is the first prospective, longitudinal investigation, exploiting detailed serial testing of predefined clinical and molecular outcome parameters (including serial CSF assessments) in a 29-year-old MNGIE patient undergoing 1 year of extensive HMD. We demonstrate that HMD only transiently restores increased serum and urine levels of thymidine and deoxyuridine, but fails to reduce CSF levels of the toxic metabolites and is ineffective to influence neurological function. These findings have direct important implications for clinical practice: They prevent a burdensome, long-term invasive, but ultimately probably ineffective procedure in future MNGIE patients.

**Electronic supplementary material:**

The online version of this article (doi:10.1186/s13023-017-0687-0) contains supplementary material, which is available to authorized users.

## Introduction

Mitochondrial neurogastrointestinal encephalomyopathy (MNGIE) is a rare, autosomal-recessive mitochondrial disorder caused by mutations in *TYMP* encoding for the thymidine phosphorylase [1, 2]. Clinically, MNGIE comprises a multisystemic syndrome of progressive leukoencephalopathy, ophthalmoparesis, demyelinating peripheral neuropathy, cachexia and gastrointestinal dysmotility [1, 2]. Biallelic carriers of *TYMP* mutations show reduced thymidine phosphorylase enzyme activity resulting in elevation of thymidine and deoxyuridine. Accumulation of these toxic metabolites leads to unbalanced intramitochondrial deoxynucleotide pools, which in turn cause site-specific mitochondrial DNA (mtDNA) depletion, multiple deletions and point mutations [1, 3, 4]. Several recent studies have demonstrated that it is indeed this nucleoside accumulation, rather than the deficiency of thymidine phosphorylase per se, that accounts for the molecular and phenotypic alterations in MNGIE [4].

Based on this pathophysiologic rationale, nucleoside reduction by dialysis has been proposed as a promising therapy in MNGIE patients [5, 6]. However, all studies so far did not measure these metabolites in cerebrospinal fluid (CSF), which is, however, the crucial compartment for CNS damage in MNGIE. Nor has any treatment study been conducted *prospectively* or with predefined quantitative outcome measures so far.

We here report a prospective, longitudinal case study with serial testing of predefined clinical and nucleoside outcome parameters (including serial CSF assessments) of a male MNGIE patient undergoing 1 year of extensive hemodialysis (HMD).

### Case report

**Fig. 1:**
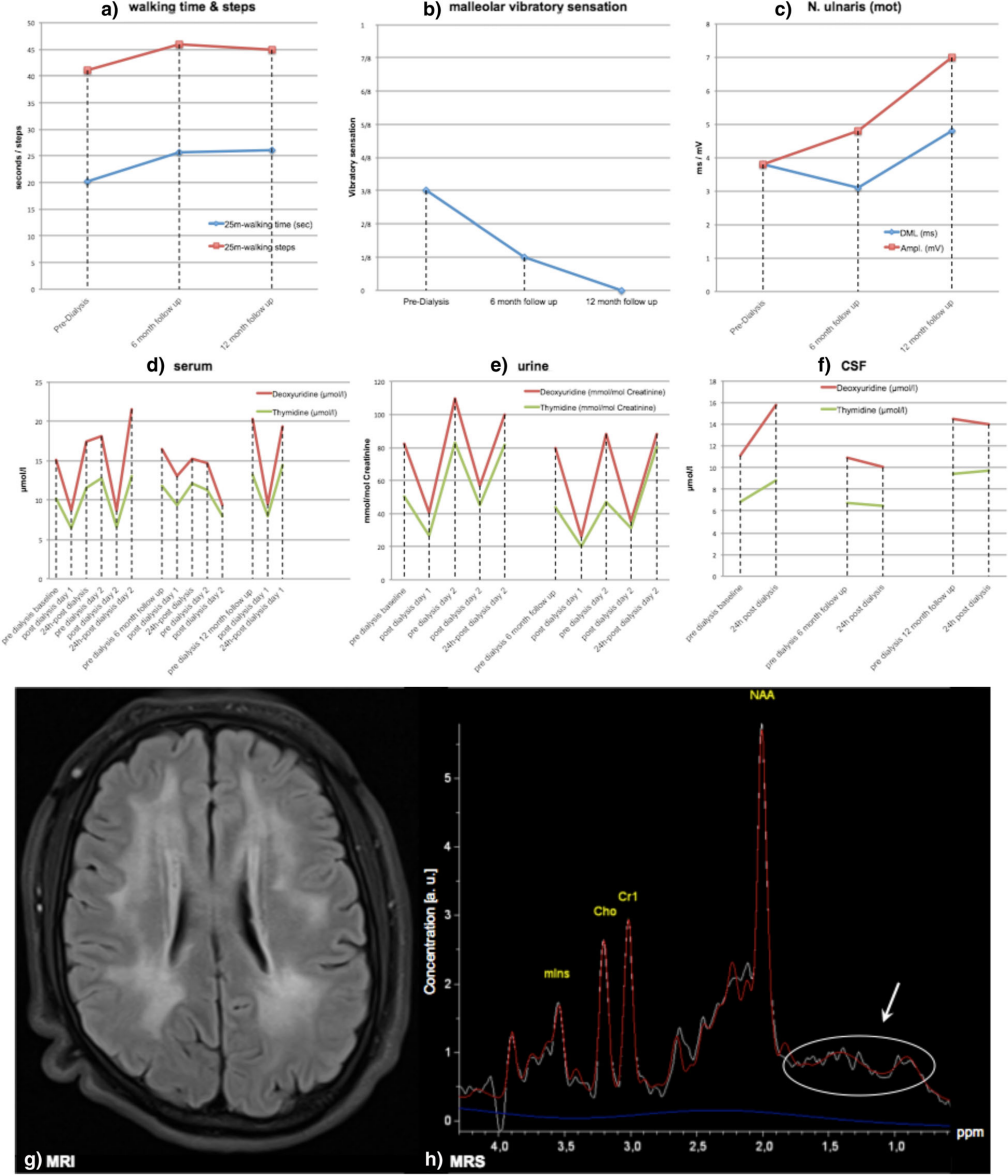
Longitudinal outcomes of clinical and molecular substrate parameters. Clinical outcomes measured by walking time and steps (**a**), malleolar vibratory sensation (**b**) and nerve conduction study of ulnar nerve (**c**) prior to the first dialysis session (“pre-dialysis”), 6- and 12-months after initiation of dialysis (“post-dialysis”). Levels of thymidine and deoxyuridine in serum (**d**), urine (**e**) and CSF (**f**) measured at baseline, 6- and 12-month follow-up each time directly before a dialysis session (“predialysis”), directly after a dialysis session (“post dialysis”) as well as 24 h after a dialysis session (“24 h–post-dialysis”). MRI showed advanced leukencephalopathy with symmetrical affection of frontal, parietal and temporal regions (**g**) and without lactate peak (*arrow*; related spectrum encircled) in MR spectroscopy (MRS) (**h**; *white curve* = measured signal, *red curve* = automatic metabolite fit, *blue curve* = 6th order polynomial representing the baseline; Abbreviations: [a.u.] = artificial unit; MI = myoinositol; NAA = N-acetyl aspartate; Cho = choline; Cr1 = creatine; DML = distal motor latency)

A 29-year-old male of Turkish decent presented with progressive gait ataxia, external ophthalmoplegia, ptosis, sensorineural hearing loss (meanwhile supplied with cochlear implants), cachexia (body weight/height/BMI before HMD: 55 kg/1.55 m/22.9 kg/m^2^; after HMD: 50 kg/1.55 m/20.8 kg/m^2^), advanced leukencephalopathy (Fig. 1g) and recurrent, diarrhea since age 18 years. Clinical examination showed short stature (149 cm), dysmorphic auricles, congenital Pes cavus and severe demyelinating sensorimotor polyneuropathy. Serum lactate (2.8–3.1 mmol/l; reference range: 0.5–2.2 mmol/l) and CSF lactate (3.5–4.6 mmol/l; reference range: 0–2.2 mmol/l) were elevated. Whole-exome sequencing (WES) revealed a homozygous *TYMP* stop mutation (c.112G > T, p.Glu38Stop) confirming the diagnosis of MNGIE (for WES methods and family pedigree, see Additional file 1).

Effectiveness of HMD was determined by the following predefined outcome measures: 25-m walking-time and -steps, vibratory sensation, nerve conduction studies and levels of thymidine and deoxyuridine in serum, urine and CSF. HMD was delivered for 12 months with an initial frequency of 3 times weekly, escalated to 4 times weekly after 6 months (for details of clinical and molecular outcome assessments and HMD parameters, see Additional file 1).

After 12-month of HMD, all clinical outcome parameters indicated progression of disease, demonstrated by worsening of SARA score (Scale for the assessment of Ataxia; 11 to 13 points), decline in MoCA score (Montréal Cognitive Assessment; 27/30 to 24/30 points) and nerve conduction measures (Fig. 1a-c). Corresponding to these these clinical observations of progressive worsening, also the subject himself did not perceive any deceleration of disease progression within the 12 months of HMD compared to the pre-HMD disease progression.

Serial testing of serum and urine levels of thymidine and deoxyuridine showed transient decreases each time after dialysis, demonstrating a reproducible *immediate* effect of HMD. However, they returned to baseline levels within 24 h and did not decrease after 6 and 12 months (Fig. 1d, e). CSF levels changed neither short-term (within 24 h) nor long term (at months 6 and 12) (Fig. 1f).

## Discussion

We present the first prospective investigation on the effectiveness of HMD in MNGIE, capturing multiple predefined outcome measures. Our results demonstrate that HMD only transiently reduces increased serum and urine levels of thymidine and deoxyuridine and is ineffective to influence clinical disease progression.

At the same time, these results provide some preliminary insights into the HMD-associated kinetics of these two metabolites in three different body fluid compartments: urine, serum and CSF. HMD was able to transiently decrease both metabolites in urine by about 50% directly after dialysis at each of the four assessment time points, showing a reproducible relative share of urinary metabolite clearance even after repeated dialysis over 12 months. However, neither the basal levels nor the maximum levels reduced over time. This urinary clearance was paralleled by a reduction of serum levels by 20–50% directly after dialysis at each of the four assessment time points (see Fig. 1d). This suggests that the kinetics of the metabolites in both compartments runs largely in parallel. Our 6-months follow-up long-term data do not provide any clear evidence for a (e.g. compensatory) increase in synthesis of metabolites in any of the two compartments (see Fig. 1d, e). Importantly, we show for the first time that HMD fails to achieve a sustained reduction of these metabolites also in the CSF compartment, which might explain the missing neurological benefit.

Our findings thus question the alleged efficacy of dialysis, which has been based so far only on retrospective case studies of peritoneal dialysis, lacking predetermined quantitative outcome measures and long-term measurement of metabolites [5, 6]. Also alternative therapeutic options in MNGIE, including liver [7] and stem cell transplantation [3], need to be critically tested by prospective studies with predefined outcome measures.

Our report has thus direct important implications for clinical practice: it prevents a burdensome, long-term invasive, but finally probably ineffective procedure in MNGIE patients.

## Additional file

Additional file 1: Whole exome sequencing, Pedigree of the index patient, Clinical and molecular substrate outcome parameters and hemodialysis parameters. (PDF 106 kb)

## Abbreviations

CNS: Central nervous system
CSF: Cerebrospinal fluid
HMD: Hemodialysis
MNGIE: Mitochondrial neurogastrointestinal encephalomyopathy
MoCA: Montréal Cognitive Assessment
MRI: Magnetic resonance imaging
mtDNA: mitochondrial DNA
SARA: Scale for the assessment of ataxia
