# Supplementary material for: Hemodialysis in MNGIE transiently reduces serum and urine levels of thymidine and deoxyuridine, but not CSF levels and neurological function
Source: Orphanet J Rare Dis. 2017 Aug 1;12:135. doi: 10.1186/s13023-017-0687-0 (PMC5540565; doi:10.1186/s13023-017-0687-0)
Supplement: Additional file 1: — Whole exome sequencing, Pedigree of the index patient, Clinical and molecular substrate outcome parameters and hemodialysis parameters. (PDF 106 kb) [file 13023_2017_687_MOESM1_ESM.pdf]

SUPPLEMENTARY MATERIAL

Supplement 1: Whole exome sequencing

Whole exome sequencing (WES) was performed on genomic DNA from two affected individuals using the SureSelect Human All Exon 50 Mb V5 kit (Agilent, Santa Clara, CA, USA) for in-solution enrichment and subsequent sequencing on a Hiseq2500 instrument (Illumina, San Diego, CA, USA). Given the autosomal recessive pattern of inheritance, we first filtered the WES data-set for potential compound heterozygous or homozygous non-synonymous or canonical splice site variants common to both affected sibs. We excluded variants with a minor allele frequency > 0.1% in an in-house database and public databases (1000 Genomes, dbSNP 142, Exome Aggregation Consortium (ExAC) Server) and searched for variants common to both affected sibs. This filtering revealed one homozygous variant c.112G>T, p.(Glu38*) in an OMIM-listed gene, *TYMP* (NM_001113756.1), which had been previously associated with clinical features of the patients. The variant was absent from 8,000 in-house exomes as well as public databases (1000 Genomes, dbSNP 142, ExAC Server (Cambridge, MA [02/2016]). This variant has already been reported asclinically relevant in a previous MNGIE family [1].

Supplement 2: Pedigree of the index patient

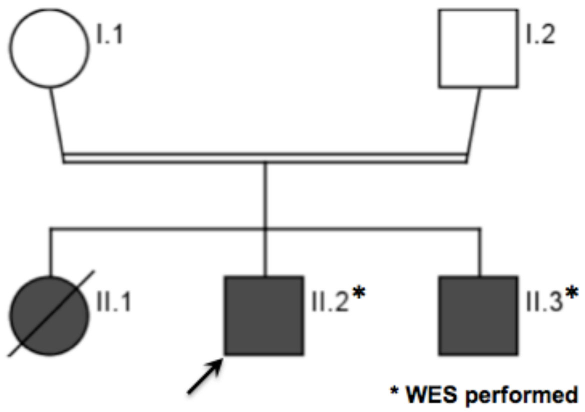

All three siblings of the family, born to consanguineous parents, were suffering from MNGIE. The sister of the index patient had already died due to bowel obstruction (subject II.1). Whole exome sequencing of the index patient (subject II.2; arrow) and his brother (subject II.3), identified a known homozygous *TYMP* stop mutation (c.112 G>T, p.Glu38*), confirming the diagnosis of MNGIE [1].

Supplement 3: Clinical and molecular substrate outcome parameters and hemodialysis parameters

Clinical outcome measures. Neuropathy was clinically assessed by the presence of areflexia, distal muscle atrophy and fasciculations, and/or impaired vibratory sensation (≤5/8). Nerve conduction studies were performed and analyzed as described previously by our group [2]. 25-meter walking test was conducted analogous to a standardized protocol previously described by Motyl et al. [3]. The steps needed for the 25-meter distance were counted as additional measure to assess gait speed and monitor changes in patients' physical function over time.

Molecular substrate outcome measures. Thymidine and deoxyuridine in urine were measured by diluting 150 µl urine with 150 µl 0.04 mM sodium acetate. 50 µl of this dilution was injected on a C18 column (Kinetex 5µm C18 150 x 4.6mm, Phenomenex). The purines and pyrimidines were eluted by HPLC (Waters Alliance e2698) by a linear gradient, starting with 100 % ammoniumacetate buffer, pH 4.75, to 95 % of an organic eluent (80% MeOH, 10 % tetrahydrofurane, 10 % acetonitrile) in 22 minutes. For measurement of plasma and CSF levels of thymidine and deoxyuridine an plasma aliquot of 350 µl or CSF was deproteinized with 100 µl 20% perchloric acid. After centrifugation 50 µl of the supernatant was injected to the column and HPLC as described above. The purines and pyrimidines were eluted over 23 minutes by a linear gradient, starting with 100 % of a 20mM potassiumdihydrogenphosphate buffer (pH 5.6) to 30 % organic eluent (100 % MeOH).

Hemodialysis parameters. Hemodialysis (HMD) was performed three times weekly, escalated to 4 times weekly after 6 months of our HMD trial, with dialysis duration of 4 hours, blood flow of 180 ml/min and dialysate flow of 500 ml/min for an optimal clearance.

Supplement references:

1. Baris, Z., et al., *Mitochondrial neurogastrointestinal encephalomyopathy (MNGIE): case report with a new mutation.* Eur J Pediatr, 2010. **169**(11): p. 1375-8.
2. Linnemann, C., et al., *Peripheral Neuropathy in Spinocerebellar Ataxia Type 1, 2, 3, and 6.* Cerebellum, 2016. **15**(2): p. 165-73.
3. Motyl, J.M., et al., *Test-retest reliability and sensitivity of the 20-meter walk test among patients with knee osteoarthritis.* BMC Musculoskelet Disord, 2013. **14**: p. 166.
